# Impact of ischemic stroke topography on early clinical outcome of basilar artery occlusion: a retrospective study

**DOI:** 10.1007/s00330-024-10755-y

**Published:** 2024-04-25

**Authors:** Moritz R. Hernandez Petzsche, Christian Maegerlein, Silke Wunderlich, Benno Ikenberg, Claus Zimmer, Jan S. Kirschke, Tobias Boeckh-Behrens, Maria Berndt

**Affiliations:** 1 Department of Diagnostic and Interventional Neuroradiology, Klinikum Rechts der Isar, School of Medicine, Technical University of Munich, Munich, Germany; 2 Department of Neurology, Klinikum Rechts der Isar, School of Medicine, Technical University of Munich, Munich, Germany

**Keywords:** Stroke, Basilar Artery, Stenosis, Diffusion-weighted imaging, Thrombectomy

## Abstract

**Objectives:**

Basilar artery occlusion (BAO) may be etiologically attributed to embolism or in situ thrombosis due to basilar stenosis (BS). Patients with BAO due to BS (BAOS) are known to have worse outcomes than patients with embolic occlusions (BAOE). BAOS occurs more proximally in the basilar artery (BA) than BAOE. We hypothesize that differing brain stem infarct patterns contribute to outcome differences between these stroke etiologies.

**Methods:**

This retrospective study includes 199 consecutive patients with BAO who received endovascular treatment at a single center. Final infarction in brain parenchyma dependent on the posterior circulation was graded semiquantitatively on magnetic resonance imaging (MRI). Associations to underlying stenosis and angiographic and clinical outcome variables were tested. The primary endpoint was early good clinical outcome (EGCO, mRS score ≤ 3 at discharge).

**Results:**

Infarct extension of the medulla oblongata (OR = 0.25; 95% CI = 0.07–0.86; *p* = 0.03), the inferior pons (OR = 0.328; 95% CI = 0.17–0.63; *p* = 0.001), the superior pons (OR = 0.57; 95% CI = 0.33–0.99; *p* = 0.046), and the occipital lobes (OR = 0.46; 95% CI = 0.26–0.80; *p* = 0.006) negatively predicted EGCO. Infarct extension for other posterior-circulation-dependent brain regions was not independently associated with unfavorable early outcomes. Patients with BAOS had more proximal occlusions and greater infarct volumes in the inferior brain stem. Successful reperfusion (mTICI 2b-3) occurred more often in patients with BAOE than in BAOS (BAOE: 131 (96.3%); BAOS: 47 (83.9%), *p* = 0.005).

**Conclusion:**

Unfavorable early outcomes in patients with BAOS may be explained by a higher likelihood of inferior brain stem infarcts and lower rates of reperfusion success.

**Clinical relevance statement:**

Basilar artery occlusion due to underlying stenosis is associated with a poorer prognosis than that caused by embolism; these results suggest that aggressive endovascular therapy, usually involving the placement of a permanent stent, may be warranted in these patients.

**Key Points:**

- *Inferior brain stem and occipital infarcts are prognostically unfavorable in basilar artery occlusion.*
- *Basilar artery occlusion due to stenosis occurs more proximally and is associated with worse outcomes.*
- *Differentiating etiologies of basilar artery occlusion may influence how aggressively treated the occlusion is.*

## Introduction

Basilar artery occlusion (BAO) is considered a life-threatening emergency and is associated with high morbidity and mortality [1–3]. The etiology of most BAOs can be broadly divided into two categories: embolism and atherosclerosis. Other rare causes of BAO, which did not play a relevant role in this study, include basilar dissection or occlusions due to basilar aneurysms [4]. Embolism may be cardio-embolic or arterial-embolic, the latter being due to a vascular pathology like atherosclerosis or dissection of a proximally located arterial vessel. Atherosclerosis of the basilar artery (BA) causes basilar stenosis (BS) and may lead to local thrombus formation due to plaque rupture. BAO due to basilar stenosis (BAOS) occurs more proximally in the BA than BAO due to embolism (BAOE) [5–7].

BAOS has been associated with a worse outcome compared to BAOE [5–7]. However, the exact mechanism of the worst outcome remains unclear.

Brain stem infarcts are largely caused by occlusion of perforating arteries from the adjacently situated large vessels: the vertebral, basilar, or posterior cerebral arteries. Differences in prognostic outcomes may be due to differing patterns of brain stem infarcts; i.e., inferior brain stem infarcts in BAOS and superior brain stem infarcts in BAOE.

In this large single-center cohort at our comprehensive stroke center, we aimed to investigate the impact of infarct topography on clinical outcomes in patients with BAOS vs. BAOE. We hypothesized that worse outcome is mediated by infarcts of the inferior brainstem due to a more proximal occlusion location in patients with BAOS.

## Materials and methods

### Study population

This retrospective, single-center study included all consecutive patients who were admitted for ischemic stroke due to acute BAO at our comprehensive stroke center between March 2008 and June 2021 (*n* = 199). During the recruitment period, approximately 2800 patients received endovascular treatment for any type of ischemic stroke at our center. Patients with angiographically verified thromboembolic material in the BA who were referred for endovascular treatment were included in this study. Part of the patient cohort has been previously described [6, 8, 9]. For 144 out of the 199 patients, post-stroke magnetic resonance imaging (MRI) was available for ischemia grading. For all analyses not requiring MRI (grading of occlusion location and clinical and baseline clinical and outcome variables), we included all consecutive 199 patients to increase the generalizability of our findings. See Fig. S1, a flowchart of subject inclusion.

### Ethical approval

Approval was obtained by the local ethics board in accordance with regional law under reference number 274/21 S-SR. Patient informed consent was waived by the ethics committee due to the retrospective nature of the study.

### Clinical parameters

Clinical and imaging data were acquired retrospectively. Clinical, demographic, outcome, and procedural data of patients were gathered. National Institutes of Health Stroke Scale (NIHSS)-certified neurologists assessed the NIHSS score at the time of admission and discharge as part of the clinical routine. Substantial neurological improvement was defined as the difference between admission and discharge NIHSS score of ≤ 8 or discharge NIHSS score of ≤ 1 [6, 8, 10, 11]. NHISS improvement was calculated by subtracting the NHISS score at discharge from the score at admission. The modified Rankin Scale (mRS) score was used to measure disability at admission, at discharge (referred to as post-treatment in the text below), and after 3 months. An early good clinical outcome (EGCO) was defined as mRS score ≤ 3 at discharge. Due to missing follow-up patient data at 3 months (valid cases *n* = 83), discharge mRS was used as the primary endpoint.

The stroke etiology was classified according to the Trial of Org 10172 in Acute Stroke Treatment (TOAST) criteria [12]. Reperfusion success of endovascular therapy was quantified based on the modified Thrombolysis in Cerebral Infarction (mTICI) scale [13] by two independent neuroradiologists.

### Grading of occlusion site, basilar stenosis, and infarct

All image-based grading was performed with the consensus of two experienced neuroradiologists.

After dividing the BA into thirds using digital subtraction angiography (DSA), the location of contrast agent discontinuation was used for grading: (1) proximal third beginning at the confluence, and including occlusions in the V4 segment with contralateral vertebral artery aplasia as previously described [6, 8]; (2) middle third, usually distal to the anterior inferior cerebellar artery; (3) distal third, the “basilar head”, see Fig. S2. Patients with V4 occlusions but without thrombotic material in the BA and without contralateral vertebral artery aplasia were not included in this study. Patients with proximal BAO may have had simultaneous V4 occlusions and patients with middle and distal BAO may have simultaneous posterior cerebral artery (PCA) occlusions.

BAOS was classified on peri-interventional DSA by detecting an underlying BS with vessel wall abnormalities and a trend to repeated thrombosis, excluding other reasons such as dissection. In three cases, classification was not possible due to failure of vessel reperfusion.

Infarct location was graded semiquantitatively on diffusion-weighted imaging (DWI). Infarct grading was performed for the following regions of interest (ROI), exactly matching the anatomical structure: the medulla oblongata, the inferior pons, the superior pons, the mesencephalon, the diencephalon, the cerebellum, and the perfusion territory of the PCA in the telencephalon, especially the occipital lobes. The inferior brain stem was defined as medulla oblongata and pons. The superior brain stem refers to the mesencephalon.

For each ROI, the following scores were distributed visually as described below based on the DWI-positive infarct volume as a percentage of the total ROI volume. The scoring was performed as a consensus reading of two expert raters (MRHP, 3 years of experience, and MB, 6 years of experience). An exemplary scoring in the superior pons can be found in Fig. S3.

0: 0% DWI volume, no infarct

1: 1–20% DWI volume, punctiform infarcts

2: 21–60% DWI volume, larger infarcts

3: 61–100% DWI volume, subtotal to total infarction

### Statistical analysis

Variables with metric and ordinal data were described as the median and interquartile range (IQR). Categorical variables were described using absolute and relative frequencies.

Metric and ordinal data were compared using the Mann–Whitney-*U*-test when comparing two groups. Metric and ordinal data were compared using the Kruskal–Wallis test when comparing three or more groups. Pearson’s Chi-Square test was used to compare categorical variables. If categorical data was distributed on a 2 × 2 crosstab, Fisher’s exact test was used. *p*-values ≤ 0.05 were considered statistically significant.

To measure the impact of infarct extension in the ROIs on EGCO, a multivariate logistic regression model was calculated including the semiquantitative ischemia scores of all ROIs. To test the validity of this model to predict good clinical outcomes, ROC analyses were performed.

Univariate associations between baseline variables and EGCO were calculated. A multivariate logistic regression model using a stepwise forward variable selection method was calculated to predict EGCO of all variables that were significantly associated with ECOG in univariate analysis. ROC analyses were performed for this regression model.

Mediation models were calculated using the commonly available PROCESS extension for SPSS using a nonparametric bootstrap approach with 5000 replication samples to obtain a 95% CI [14, 15].

## Results

### Baseline characteristics, clinical, and angiographic outcomes

This study includes 199 patients with BAO. The median age of patients included in this study was 75 years (IQR = 63–82) and 115 (58%) were male. In *n* = 3, no etiological classification was feasible on DSA. Of the rest (*n* = 196), 139 (71%) had no underlying BS, and 57 (29%) had an underlying BS.

**Fig. 1:**
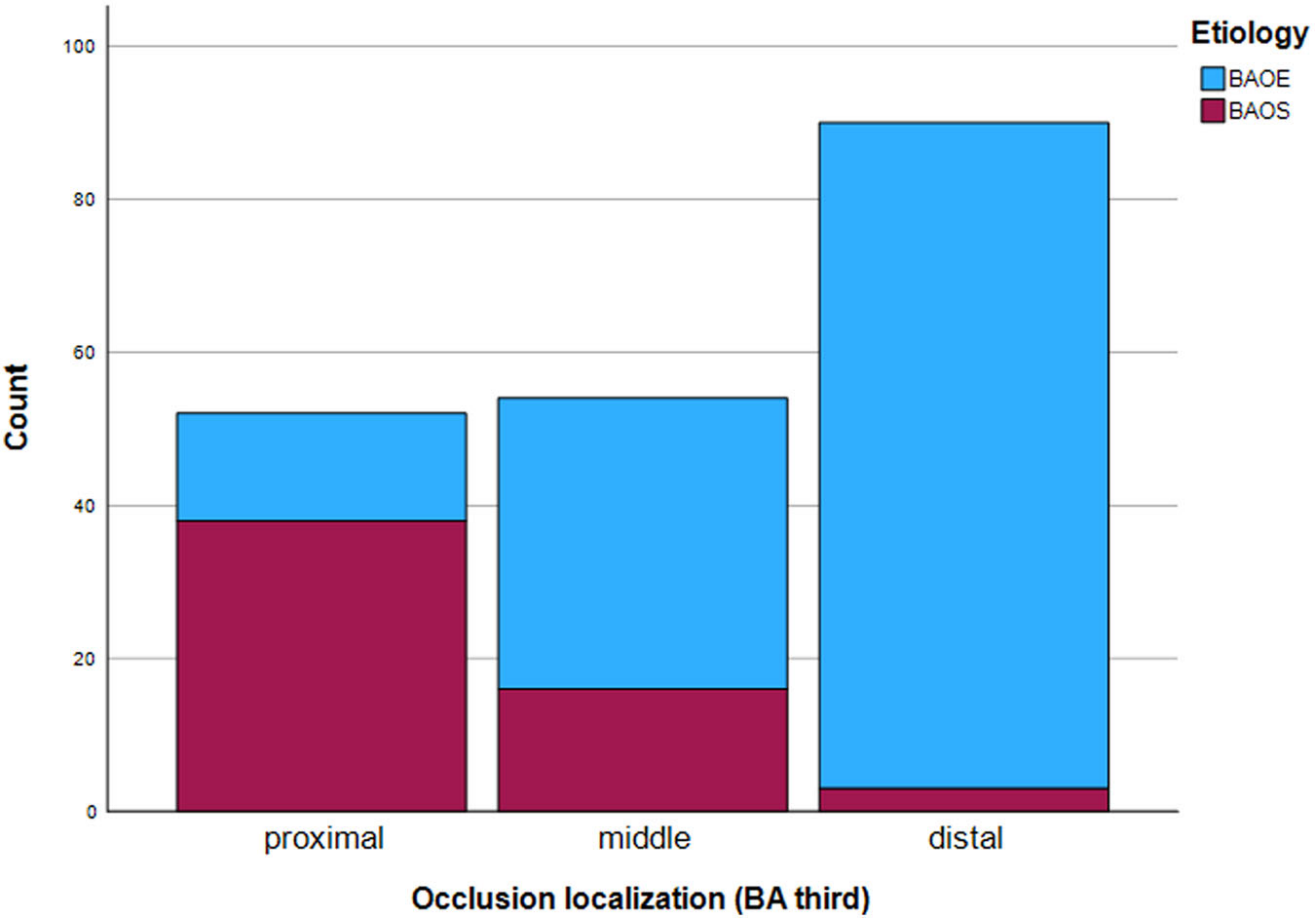
Absolute count of occlusion location in BA thirds based on underlying etiology (BAOE vs. BAOS). BAOS was usually located more proximally, preferentially in the proximal third of the BA. BAOE was more likely to occur in the distal BA, usually in the distal third (basilar head)

BAOS was significantly more likely to occur in the proximal BA than BAOE, *p* < 0.001 (Fig. 1). Patients with BAOS showed significantly higher post-treatment disability compared to patients with BAOE (median mRS = 5, IQR = 4–6 in the BAOS group; median mRS = 4, IQR = 2–5 in the BAOE group; *p* = 0.001). Similarly, significantly higher post-treatment disability was observed in patients with more proximal occlusion (proximal third median mRS = 5, IQR = 4–6; middle third median mRS = 4.5, IQR = 3–5.75; and distal third median mRS = 4, IQR = 1–5; *p* = 0.008). There was a non-significant tendency toward a better outcome in patients with more distal occlusions when considering only patients with BAOE (Fig. S4A) and only BAOS (Fig. S4B). Failure to reach statistical significance in these analyses is likely due to too few cases of proximal occlusion in BAOE and distal occlusion in BAOS. Rates of complete reperfusion (mTICI 3) and successful reperfusion (mTICI 2b or higher) were significantly lower in patients with BAOS (mTICI 3 = 99 (72.8%) in BAOE and 32 (57.1%) in BAOS, *p* = 0.04; mTICI 2b or higher = 131 (96.3%) in BAOE and 47 (83.9%) in BAOS, *p* = 0.005). However, after excluding patients who did not undergo stenting of the BA (*n* = 12), there was no significant difference in TICI scores between patients with BAOS vs. BAOE. Table 1 gives an overview of the baseline and outcome characteristics of all patients and patients divided into groups based on stroke etiology (BAOS vs. BAOE) and location of BAO in BA thirds.

**Table 1:** Baseline and outcome characteristics

	All	BAOS	BAOE	*p*	All	Proximal occlusion	Middle occlusion	Distal occlusion	*p*
*n*	196	57 (29.1%)	139 (70.9%)		199	53 (26.6%)	54 (27.1%)	92 (46.2%)	
Missing	3				0				
Baseline and procedural characteristics									
Age, years, median (IQR)	75 (63–82)	72 (61.5–79)	76 (63–82)	0.155	75 (63–82)	72 (60–79)	74 (59–82)	78 (68.4*–*83)	**0.026**
Male sex, *n*, (%)	112 (57.1%)	37 (64.9%)	75 (54.0%)	0.204	115 (57.8%)	34 (64.2%)	28 (51.9%)	53 (57.6%)	0.436
mTICI									
0–2a, *n*, (%)	14 (7.3%)	9 (16.1%)	5 (3.7%)		17 (8.5%)	7 (13.5%)	6 (11.3%)	4 (4.4%)	
2b, *n*, (%)	47 (24.5%)	15 (26.8%)	32 (23.5%)	0–2a vs. 2b/3: **0.005**	47 (23.6%)	9 (17.3%)	17 (32.1%)	21 (23.3%)	0–2a vs. 2b/3: 0.136
3, *n*, (%)	131 (68.2%)	32 (57.1%)	99 (72.8%)	**0.041**	131 (65.8%)	36 (69.2%)	30 (56.6%)	65 (72.2%)	0.154
Procedure time (min, median/IQR)	60 (31–104)	101.5 (55.5–140)	45 (26.75–85)	**0.000**	60 (31–104)	85 (49–135)	69 (35–118)	45 (25–73)	**0.000**
Onset to groin time (min, median/IQR)	270 (195–390)	305 (224.25–480)	260 (182.5–360)	0.074	270 (195–390)	300 (240–415)	261 (181.25–372.5)	252 (185.25–390)	0.223
No. of passes (median/IQR)	2 (1–3)	2 (1–3)	2 (1–3)	0.108	2 (1–3)	2 (1–3)	2 (1–3)	1 (1–2)	0.073
Success of aspiration only, *n* (%)	56 (29.0%)	6 (10.5%)	50 (36.8%)	**0.000**	56 (28.1%)	8 (15.1%)	19 (35.8%)	29 (32.6%)	**0.034**
Preinterventional intravenous tPA, *n* (%)	77 (39.3%)	19 (33.3%)	58 (41.7%)	0.334	77 (38.7%)	20 (37.7%)	19 (35.2%)	38 (41.3%)	0.754
BA stent implantation, *n* (%)	NA	45 (78.9%)	NA	NA	NA	NA	NA	NA	NA
Pre-treatment NIHSS, median (IQR)	13 (6.75–22)	14 (7–22.75)	13 (7–22)	0.630	13 (6.75–22)	16 (7–24)	13 (7–22)	12 (6–20.25)	0.436
Outcome									
Post-treatment NIHSS, median (IQR)	10 (2.75–42)	15 (8–42)	6 (2–20)	**0.001**	10 (2.75–42)	12.5 (6.5–42)	11 (3–42)	5 (1–18.5)	**0.017**
Substantial neurological improvement, *n* (%)	63 (34.4%)	14 (25.9%)	49 (38.0%)	0.128	63 (31.7%)	16 (32%)	12 (23.5%)	35 (41.2%)	0.103
NIHSS Improvement, median (IQR)	2 (−2.0–8.75)	1 (−24.0–6.5)	3 (0.0–10.0)	**0.007**	2 (−2.0–8.0)	1.5 (−18.0–8.75)	1 (−2.0–6.0)	3 (0.0–10.0)	0.463
mRS post-treatment (*n* = 190)	4 (2–5.25)	5 (4–6)	4 (2–5)	**0.001**	4 (2–5.25)	5 (4–6)	4.5 (3–5.75)	4 (1–5)	**0.008**
mRS 0–3 (early good clinical outcome), *n* (%)	70 (37.4%)	10 (18.2%)	60 (45.5%)	**0.000**	71 (35.7%)	11 (21.2%)	18 (34.6%)	42 (48.8%)	**0.004**
mRS > 3, *n* (%)	117 (62.6%)	45 (81.8%)	72 (54.5%)		119 (59.8%)	41 (78.8%)	34 (65.4%)	44 (51.2%)	
mRS after 3 months, median (IQR) (*n* = 83)	4 (1–6)	4 (1–6)	3 (1–6)	0.234	4 (1–6)	4 (3–6)	4 (1–6)	2.5 (1–6)	0.345

Bold values indicate statistical significance *p* < 0.05

### Effect of BS and occlusion location on ischemic territory

**Fig. 2:**
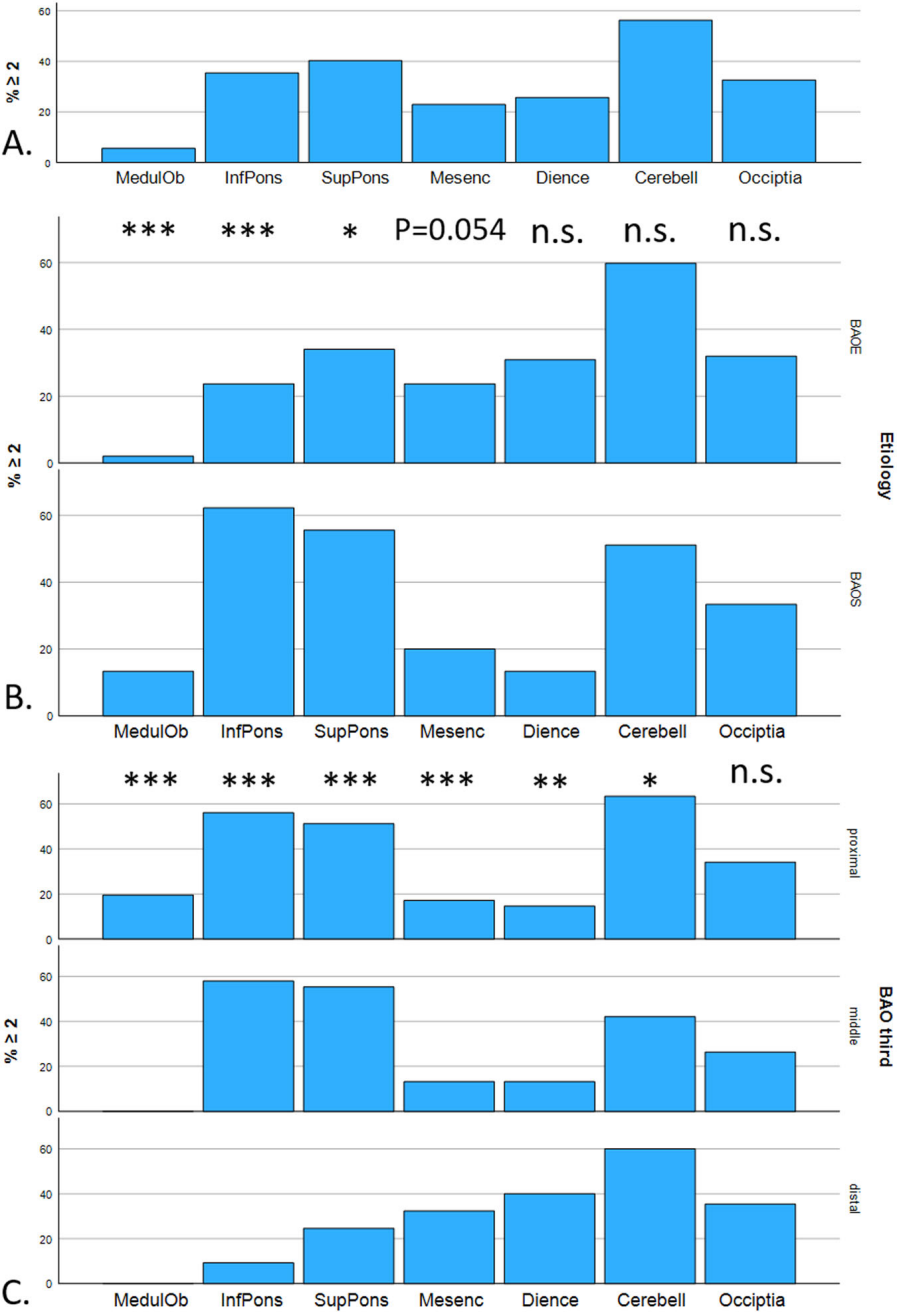
Visual semiquantitative volumetric analysis of DWI positive ischemic territory. Each ROI is plotted along the *x*-axis. The *y*-axis shows the percentage of the cohort with an ischemia score of ≥ 2. **A** Percentage of the entire cohort with ischemia scores of ≥ 2 for each ROI. **B** Percentage of the cohort with ischemia scores of ≥ 2 comparing the BAOE and BAOS groups. Statistical comparisons of ischemia scores were performed using the Mann–Whitney-*U*-test. **C** Percentage of the cohort with ischemia scores of ≥ 2 comparing occlusions in the proximal, middle, and distal third of the BA. Statistical comparisons in ischemia scores were performed using the Kruskal–Wallis test. n.s. not statistically significant, *p < 0.05, **p < 0.01, and ***p < 0.001

Of all included patients, 144 patients (72%) received a post-stroke MRI adequate for the characterization of ischemic territory. Semiquantitative volumetric analysis of DWI-positive ischemic territory was performed as described above. Figure 2A shows the likelihood of ischemia scores ≥ 2 for each ROI in all patients. Ischemia grading revealed significantly greater ischemia likelihood in the medulla oblongata and the pons in patients with BAOS in comparison to patients with BAOE (e.g.: ischemia score ≥ 2 in the medulla: 2.1% in BAOE and 13.0% in BAOS; *p* < 0.001) (Fig. 2B). No significant difference was observed in ischemia scores of the remaining ROIs, however, ischemia likelihood of the mesencephalon and diencephalon were tendentially greater in BOAE.

After dividing cases based on occlusion location, proximal occlusion showed peak ischemia likelihood in the inferior pons, whereas ischemia likelihood peaked in the mesencephalon and diencephalon in patients with distal occlusions, see Fig. 2C. There was higher ischemia likelihood in the cerebellum in patients with proximal and distal BA occlusions compared to patients with occlusions in the middle BA third.

### Impact of ischemic pattern on early clinical outcome

**Fig. 3:**
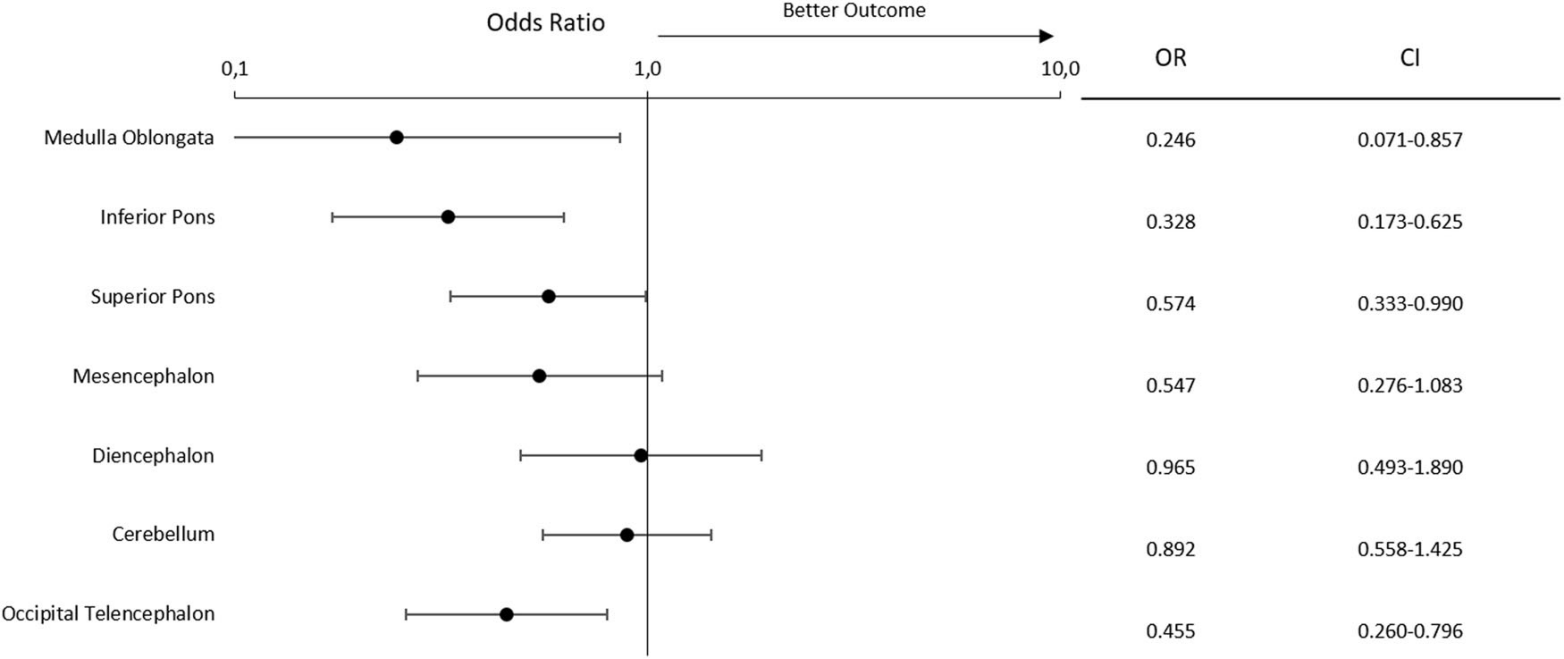
Forest plot based on a multivariate logistic regression analysis for early good clinical outcome (mRS ≤ 3). Semiquantitative volumetric ischemia scores in each ROI dependent on the posterior circulation were input as independent variables. The odds ratio (OR) and the 95% confidence interval (CI) for each ROI are displayed on the right

Only ischemia scores of the medulla oblongata (OR = 0.25; 95% CI = 0.07–0.86; *p* = 0.03), the inferior pons (OR = 0.328; 95% CI = 0.17–0.63; *p* = 0.001), the superior pons (OR = 0.57; 95% CI = 0.33–0.99; *p* = 0.046), and the occipital lobes (OR = 0.46; 95% CI = 0.26–0.80; *p* = 0.006) were independently negatively associated with EGCO. Ischemia scores in the mesencephalon, the diencephalon, and the cerebellum did not independently predict EGCO (Fig. 3). For a ROC analysis of this regression model, see Fig. S5A.

### Impact of baseline data on early clinical outcome

Primary, univariate non-adjusted analyses were performed for various variables to test their association with EGCO. Factors such as age, occlusion site, underlying BS, diabetes as a risk factor, complete recanalization (TICI 3), and procedure time were significantly associated with EGCO, see Table S1.

The variables age (OR = 0.96; 95% CI = 0.94–0.99; *p* = 0.005), occlusion location (OR = 1.86; 95% CI = 1.17–2.96; *p* = 0.009), diabetes (OR = 0.13; 95% CI = 0.04–0.45; *p* = 0.002), and procedure time (OR = 0.98; 95% CI = 0.98–0.99; *p* = 0.008) were independently associated with EGCO, see Table 2. A ROC curve of this regression study is found in Fig. S5B.

**Table 2:** Factors predicting early good clinical outcome (mRS score ≤ 3)

	Regression coefficient *β*	*p*	Odds ratio (95% CI)
Occlusion location (BA thirds)	0.619	0.009	1.856 (1.165–2.958)
Procedure time	‒0.011	0.008	0.981 (0.981–0.997)
Age	‒0.039	0.005	0.962 (0.936–0.988)
Diabetes	‒2.069	0.002	0.126 (0.035–0.454)

Excluding procedure time due to collinearity with mTICI as well as with BAOS and excluding occlusion location due to collinearity with BAOS from multivariate analyses, complete reperfusion (mTICI 3) (OR = 2.24, 95% CI = 1.05–4.78), and BAOS (OR = 0.30, 95% CI = 0.13–0.68) showed a significant association to EGCO, see Table S2. A ROC curve of this regression study is found in Fig. S5C.

### Role of ischemic pattern for outcome in BAO with BS

**Fig. 4:**
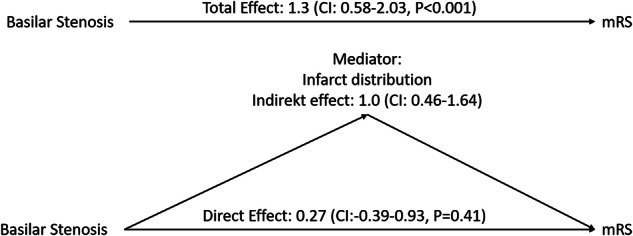
Mediation model with BS as the causal (independent) variable, post-treatment mRS as the outcome (dependent) variable, and the semivolumetric infarction scores for all investigated brain areas as mediator variables

A mediation model with BS as the causal (independent) variable, post-treatment mRS as the outcome (dependent) variable, and the semivolumetric infarction scores for all investigated brain areas, as mediator variables were calculated. The total effect of BS on post-treatment mRS was 1.3 (95% CI = 0.58–2.03, *p* < 0.001). The bootstrapped 95% CI for the indirect effect was different from zero, indicating the infarct distribution mediates the relationship between BS and clinical outcome. The indirect effect of ischemic extension on the outcome considering the ischemia scores for all ROIs was 1.0 (95% CI = 0.46–1.64), whereas amongst all ROIs, only ischemia of the medulla oblongata (effect = 0.31, 95% CI = 0.08–0.60), the inferior pons (effect = 0.61, 95% CI = 0.22–1.08), and the superior pons (effect = 0.27, 95% CI = 0.04–0.61) individually significantly mediated the effect of BS on outcome, see Table S3. The direct effect of BS on mRS lost statistical significance (direct effect: 0.27; CI: –0.39–0.93; *p* = 0.41) (Fig. 4).

## Discussion

This large, single-center study including 199 patients treated for BAO between 2008 and 2021 yields two main results: firstly, the early outcome in patients with BAO is dependent on the degree of infraction of the inferior brain stem and the occipital lobes. This finding partly explains a worse outcome in BAOS, where more proximal occlusions lead to inferior brain stem infarcts. Second, a worse prognosis of BAOS is also partly due to diminished angiographic outcome.

### Functional eloquence of brain stem tissue

The brain stem acts as a crucial conduit for major ascending and descending fiber tracts governing motor, sensory, and vegetative functions. It houses nuclei of cranial nerves and circuits for central respiratory and circulation control, compressing numerous vital neuroanatomical structures into a compact space. Even small, punctiform brain stem infarcts can result in significant neurological deficits. The functional eloquence of brain stem tissue elucidates the unfavorable outcomes observed in BAO, irrespective of its etiology. In this study, we use regression analysis to show ischemia in the medulla oblongata and pons as well as ischemia in the territory of the PCA in the telencephalon independently predicting bad early outcomes. Ischemia in the mesencephalon and diencephalon did not independently predict early outcomes. The brain stem receives its blood supply largely from small perforating arteries stemming directly from the BA. We show that occlusion of the proximal BA leads to inferior brain stem infarcts whereas thrombosis of the basilar apex predisposes to infarcts of the superior brain stem like the mesencephalon and thalamus. This is consistent with perforator ischemia at the location of the thrombus with little potential for collateral flow, causing adjacent tissue infarction.

In the context of BAO, where infarct patterns are complex and usually include more than one anatomical brain stem region, we show that the overall drivers of outcome are infarct volumes in the inferior brain stem (pons and medulla oblongata) as opposed to the mesencephalon and thalamus. The downward shift of maximum infarct extension and likelihood in the brain stem, as shown in this study, from superior (mesencephalon, diencephalon in embolic occlusions) to inferior (pons, medulla oblongata in stenotic occlusions), therefore, likely partially explains the differences in outcome observed between the two groups.

Patients with isolated superior brain stem infarcts may also suffer from disastrous outcomes, even without concomitant infarcts in the inferior brain stem, this has been well-documented in literature [16, 17] and is frequently observed in clinical routine. However, considering the overall infarct pattern within BAO, a shift from the superior to the inferior brain stem is prognostically unfavorable, a motif that was seen in the entire cohort and as a tendency for both the embolic and stenotic subgroups individually.

Infarcts in the occipital lobes also predicted worse early outcomes in regression analysis. This is possibly due to a risk of tissue swelling and mass effect-related complications in this comparatively large area of vascular territory. In a mediation analysis, we show that infarct distribution significantly mediates the relationship between BS and outcome. Similar to the regression analysis, mediation analysis showed significant mediation of BS on mRS only for ischemic volumes in the medulla oblongata and pons.

### Worse angiographic outcome in patients with basilar stenosis

We showed that patients with BAOS had significantly worse angiographic outcomes as measured by mTICI than patients with BAOE. After excluding procedure time and occlusion location due to collinearity with reperfusion success and presence of BS, respectively; both reperfusion success and presence of BS were significantly associated with outcome in multivariate analysis.

However, excluding patients who did not receive stenting in the BAOS group (*n* = 12), no differences in angiographic outcome were observed between patients with BAOS and patients with BAOE.

### Implications

In the BASILAR registry study, as well as the recently published ATTENTION and BAOCHE trials, patients with proximal occlusions profited more from interventional treatment than patients with mid- or distal occlusions [18–20]. These findings are likely due to a higher incidence of stenotic occlusions in the proximal BA third, where medical therapy including intravenous thrombolysis has little therapeutic effect. However, the presence or absence of underlying stenosis was not explicitly recorded in these studies.

Due to differences in stroke mechanism and etiology, BAOS should be treated as an entirely different disease entity. Stenotic occlusions, with their propensity to cause inferior brain stem infarcts and overall diminished angiographic outcome, should be afforded aggressive interventional therapy with a low threshold to perform stenting if re-occlusion is deemed likely. Previous studies have aimed to identify a patient’s stenotic BAO using admission CT data, facilitating pre-intervention workflows like planning for a longer procedure time, possible stenting, and antithrombotic therapy [6, 9]. Further studies are required to establish appropriate treatment approaches for patients with stenotic BAO and prospective studies that are conducted in the future should track and analyze the sub-population of BAOS to determine their impact on overall trial outcome.

### Limitations

This study is limited by its retrospective and single-center design. Not all patients with BAO included in this study later received MRI in a clinical routine that was required for visual analysis, possibly leading to a slight selection bias. Follow-up data was limited in this patient cohort, mRS after three months was not sufficiently available to use as a primary end-point. MRS at hospital discharge was used alternatively in this study, possibly limiting the comparability of results to other studies. The use of mTICI classification may be another limitation as it is limited in its classification of residual side branch occlusion after BAO. As this is particularly relevant for ischemic patterns, it should be the focus of further studies.

## Conclusion

In BAO, infarct extension in the inferior brain stem (medulla oblongata/pons) and the occipital lobes determine the overall outcome. The functional eloquence of the inferior brain stem partially explains reduced outcome in BAOS, where occlusions occur more proximally in comparison to BAOE. Moreover, the angiographic result, assessed through mTICI, is compromised in individuals with BAOS, particularly among those who do not undergo stenting, leading to a further decline in clinical outcome.

## Supplementary information

Electronic Supplementary Material

## Abbreviations

BA: Basilar artery
BAO: Basilar artery occlusion
BAOE: Basilar artery occlusion due to embolism
BAOS: Basilar artery occlusion due to basilar stenosis
BS: Basilar stenosis
DSA: Digital subtraction angiography
DWI: Diffusion-weighted imaging
EGCO: Early good clinical outcome, mRS at discharge ≤ 3
IQR: Interquartile range
MRI: Magnetic resonance imaging
mRS: Modified Rankin Scale
mTICI: Modified treatment in cerebral infarction
NIHSS: National Institutes of Health Stroke Scale
PCA: Posterior cerebral artery
