# Supplementary material for: Impact of ischemic stroke topography on early clinical outcome of basilar artery occlusion: a retrospective study
Source: Eur Radiol. 2024 Apr 25;34(10):6796–804. doi: 10.1007/s00330-024-10755-y (PMC11399215; doi:10.1007/s00330-024-10755-y)
Supplement: Supplementary file 1 — Electronic Supplementary Material [file 330_2024_10755_MOESM1_ESM.pdf]

Impact of ischemic stroke topography on early clinical outcome of basilar artery occlusion: a retrospective study

Electronic Supplementary Material (ESM)

Eur Radiol (2024) Hernandez Petzsche MR, Maegerlein C, Wunderlich S et al.

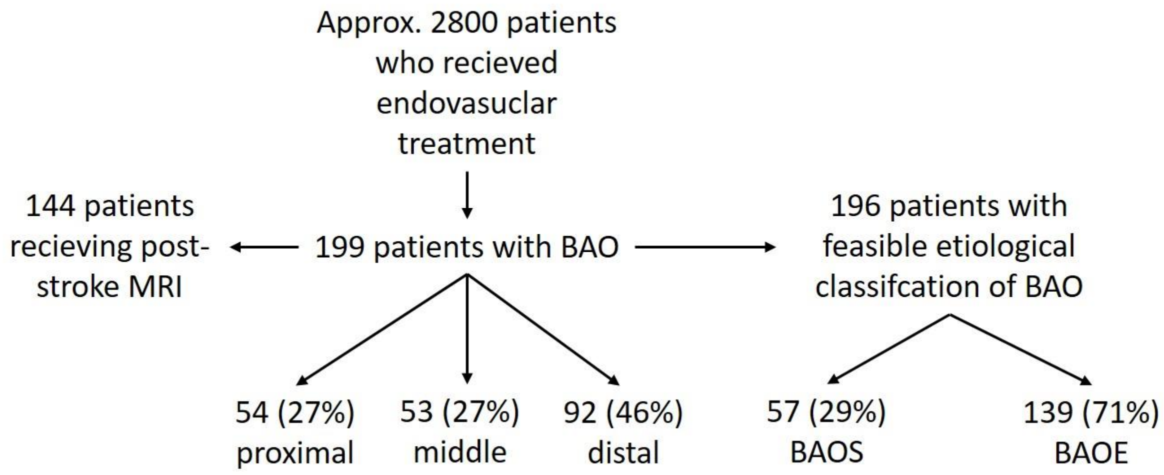

Figure S1. Flow-chart of patient inclusion

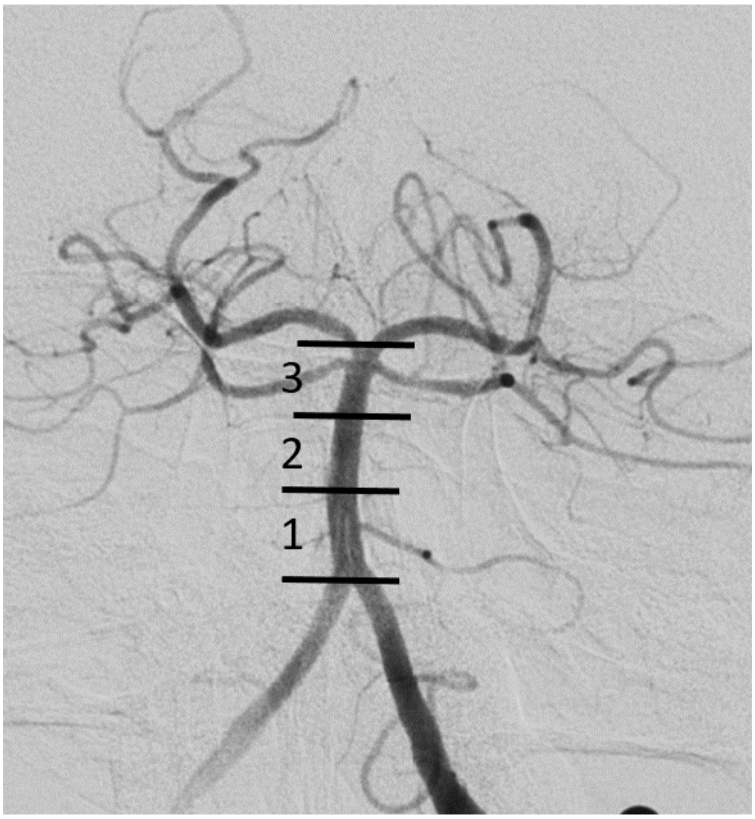

Figure S2. Grading of occlusion location was based on the most proximal

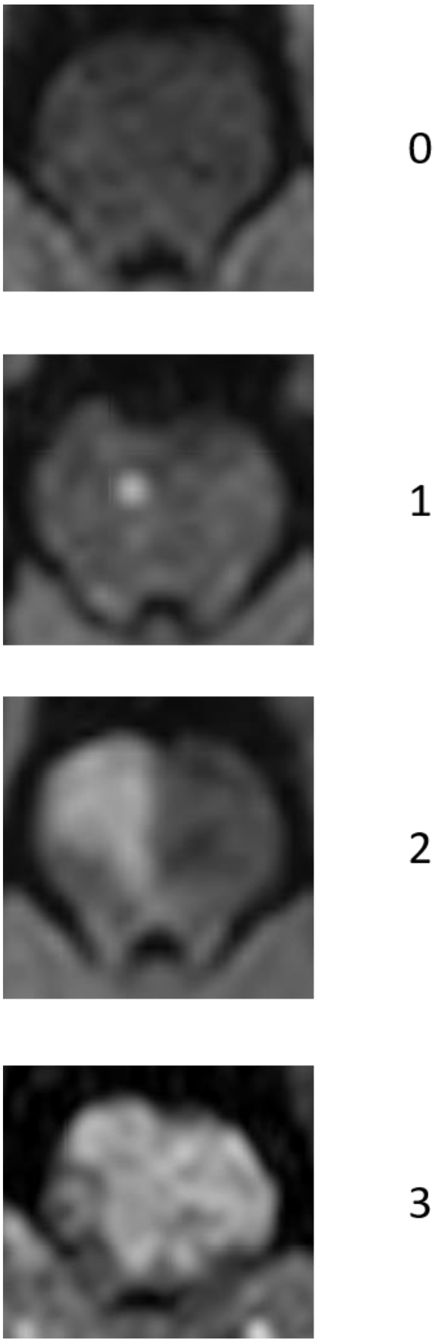

Figure S3. Examples of infarct scores in the superior pons on diffusion weighted imaging.

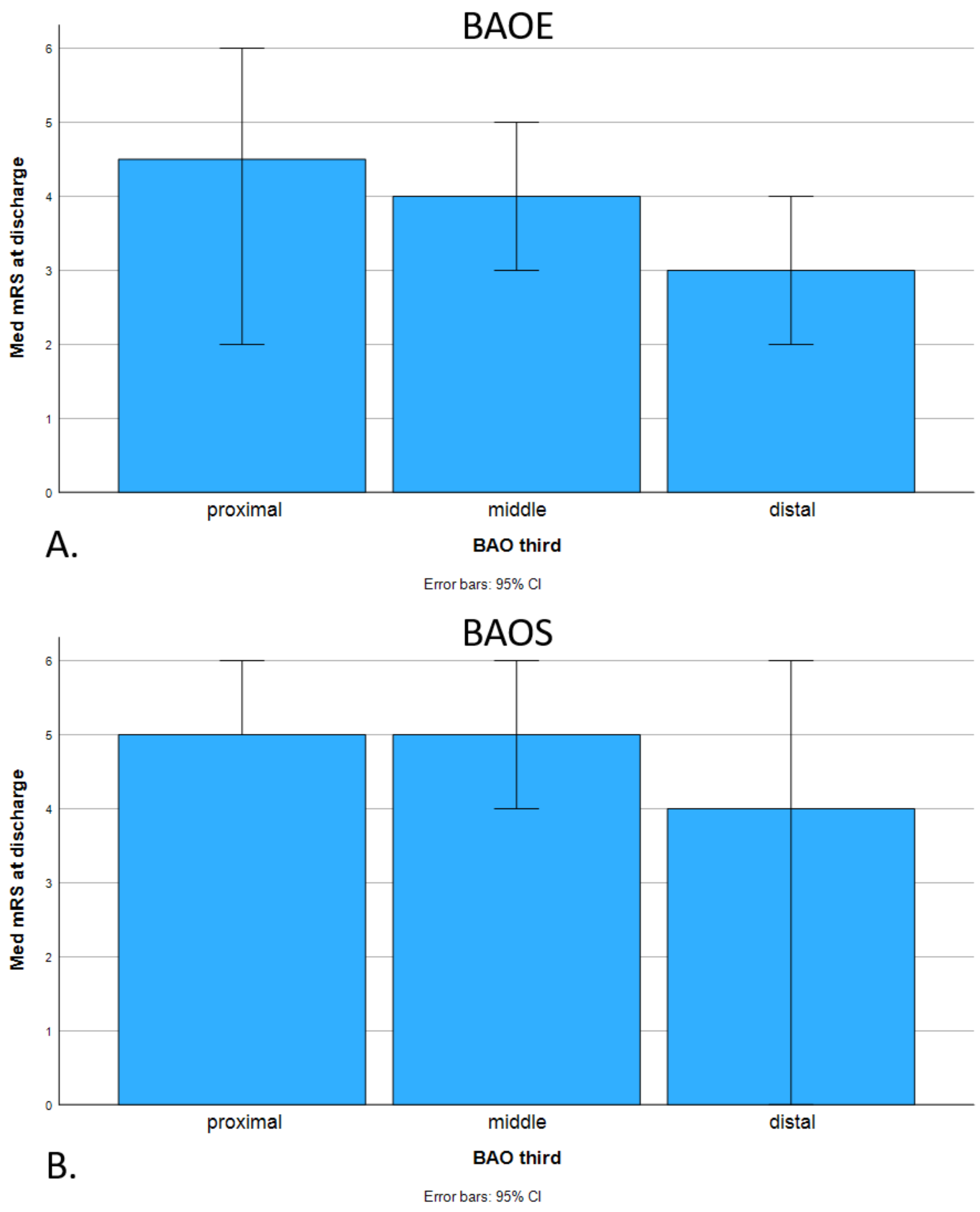

Figure S4. Median mRS scores at discharge the sub-cohort of embolic-only occlusions (A.) and stenotic only occlusions (B.)

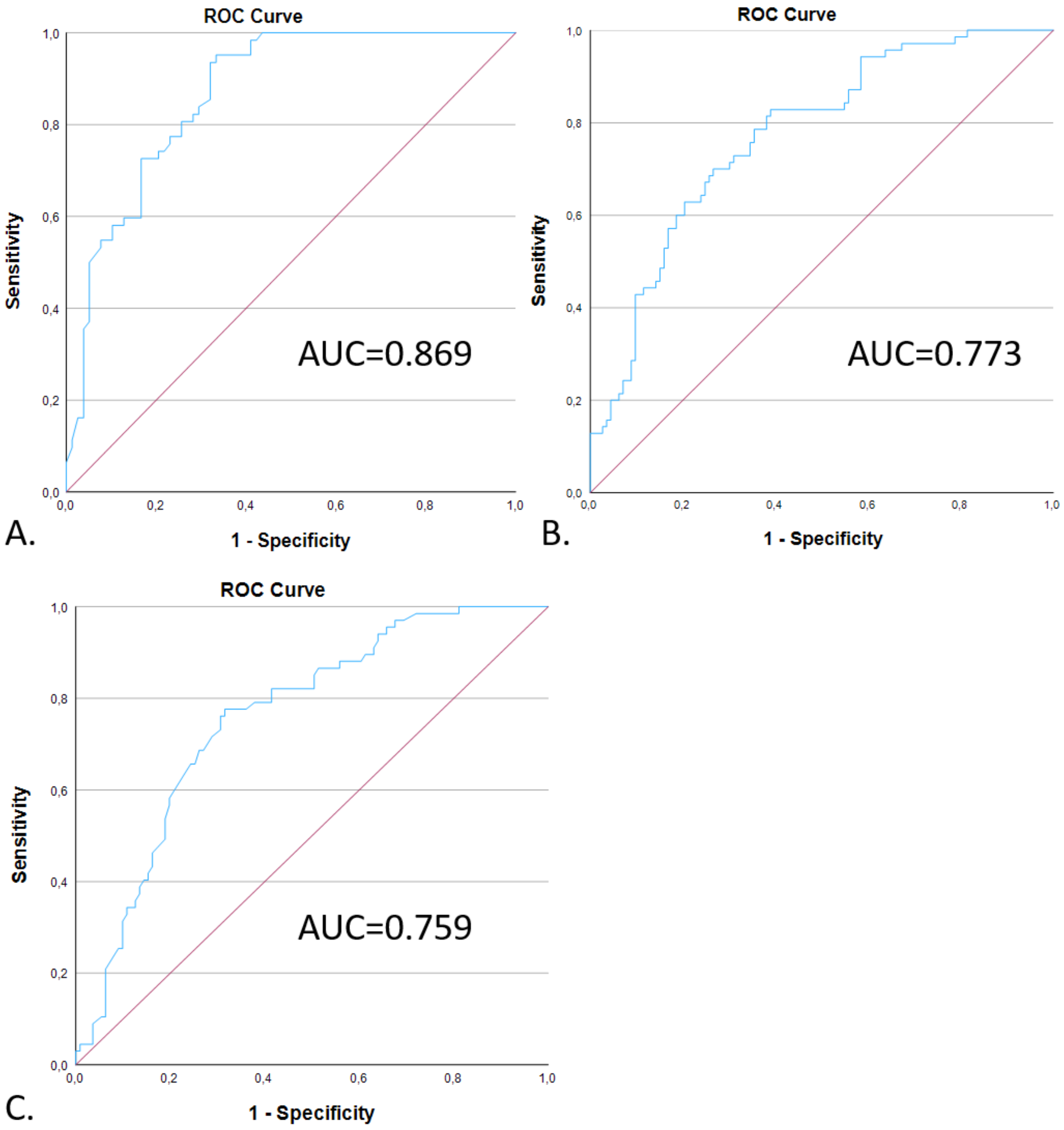

Figure S5. ROC-Analyses.

ROC-Curve of the regression model summarized in Figure 3, examining the effect of infarct extension in various brain regions of the posterior circulation on outcome; the AUC is 0.869 (A.). ROC-Curve of the regression model that is summarized in Table 2 of the manuscript measuring the effect of clinical variables on outcome, that were associated with good clinical outcome in univariate analyses; the AUC is 0.773 (B.). ROC-Cure of the regression model that is summarized in Table S2, measuring the effect of clinical variables on outcome after excluding procedure time and BS due to collinearity; the AUC is 0.759 (C.).

	Values (percentage for dichotomous variables, median/IQR for parametric varialbes)	Univariate analyses (p-values)
**Baseline characteristics**		
Age, y, median (IQR)	75 (63 - 82)	**0.03**
Male sex, n, (%)	115 (57.8%)	0.45
NIHSS (median (IQR)) Pre-treatment	13 (6.75 - 22)	**<0.0001**
Occlusion site (prox, med, dist, %)	26.6%, 27.1%, 46.2%	**0.004**
Underlying basilar stenosis, n (%)	57 (29.1%)	**0.0004**
Peri-interventional intravenous tPA, n (%)	77 (38.7%)	0.17
**Risk Factors**		
Diabetes Mellitus	34 (17.1%)	**0.0001**
Atrial fibrillation	76 (38.2%)	0.06
Hypertension	144 (72.4%)	0.40
Previous TIA or Stroke	47 (23.6%)	0.86
**Procedural variables**		
Successful recanalization (TICI 2b/3)	178 (91.3%)	0.11
Complete recanalization (TICI 3)	131 (65.8%)	**0.01**
Procedure time (min, median/IQR)	60 (31 - 104)	**0.0002**
Time from symptom onset to groin puncture (min, median/IQR)	270 (195 - 390)	0.61

Table S1. Patient characteristics and factors associated with good clinical outcome.

Baseline demographic, clinical and interventional data for all patients and univariate analyses to test for a difference between the groups in good clinical outcome (mRS 0-3). Mann-Whitney-U tests for non-parametric variables, Fisher's exact test for dichotomous categorical variables. Abbreviations: mRS modified Rankin Scale, NIHSS National Institute of Health Stroke Scale, TICI modified thrombolysis in cerebral infarction, tPA tissue Plasminogen Activator, IQR interquartile range

	Regression Coefficient β	P=	Odds Ratio (95% CI)
Age	-0.032	0.015	0.969 (0.944-0.994)
Complete recanalization (mTICI 3)	0.806	0.037	2.240 (1.049-4.781)
Diabetes	-2.020	0.002	0.133 (0.037-0.471)
Underlying BA Stenosis	-1.214	0.004	0.297 (0.130-0.676)

Table S2. Factors predicting good clinical outcome (mRS Score ≤3) excluding procedure time from multivariate analyses due to a high collinearity with mTICI and occlusion location due to a high collinearity with BS.

Indirect Effect of Infarct Distribution	Effect	Lower 95% C.I.	Upper 95% C.I.
TOTAL*	1.0322	0.4597	1.6411
Medulla oblongata*	0.3062	0.0834	0.6004
Inferior Pons*	0.6105	0.2184	1.0781
Superior Pons*	0.2673	0.0423	0.611
Mesencephalon	-0.0177	-0.1499	0.0704
Diencephalon	-0.0666	-0.2446	0.0408
Cerebellum	-0.0477	-0.1997	0.0993
Occiptial Telencephalon	-0.0199	-0.186	0.1471

Table S3. Mediation model with BS as the causal (independent) variable, post-treatment mRS as the outcome (dependent) variable, and the semivolumetric infarction scores for all investigated brain areas as mediator variables. * indicating statistical significance.
